# Comparison of Sodium Zirconium Cyclosilicate to Calcium Polystyrene Sulfonate for Acute Hyperkalemia Among Hospitalized Elderly Patients

**DOI:** 10.7759/cureus.71750

**Published:** 2024-10-18

**Authors:** Junpei Komagamine, Satsuki Yoshihara, Tomohiro Kurihara

**Affiliations:** 1 Emergency and Critical Care Medicine, National Hospital Organization Tokyo Medical Center, Meguro, JPN; 2 Internal Medicine, National Hospital Organization Tokyo Medical Center, Meguro, JPN

**Keywords:** calcium polystyrene sulfonate, elderly people, emergency medicine and trauma, severe hyperkalemia, sodium zirconium cyclosilicate

## Abstract

Background

Our aim was to investigate the effect of sodium zirconium cyclosilicate (SZC) on acute hyperkalemia in hospitalized elderly patients.

Methods

A retrospective observational study was conducted. All consecutive patients aged 65 years or older who were hospitalized in our hospital between January 2020 and September 2023 were screened. Patients with serum potassium greater than 5.0 mEq/L and those who were prescribed either SZC or calcium polystyrene sulfonate (CPS) during hospitalization were included. The primary outcome was the change in serum potassium within 24 hours from baseline. Patients who were prescribed SZC and those who were prescribed CPS were compared by the Mann-Whitney test.

Results

A total of 87 hospitalized elderly patients with acute hyperkalemia were included. The median age of the patients was 81 years (IQR 75 to 86); 64 (73.6%) were men, and 64 (73.6%) had chronic kidney disease. The median serum potassium concentration was 6.0 mEq/L (IQR 5.7 to 6.4). Of those, 53 patients received SZC, and 35 patients received CPS. While SZC reduced the serum potassium concentration from 6.1 mEq/L to 5.0 mEq/L, CPS reduced the potassium concentration from 5.9 mEq/L to 5.3 mEq/L within 24 hours. Compared with CPS, SZC significantly reduced potassium (*p* = 0.003).

Conclusions

Compared with CPS, SZC might reduce serum potassium more among hospitalized elderly patients with hyperkalemia. Further studies are warranted to determine the role of SZC for acute hyperkalemia in acute care settings.

## Introduction

Hyperkalemia, defined as a serum potassium level greater than 5.0 mEq/L, is an electrolyte abnormality that can lead to fatal arrythmias if left untreated. The prevalence of hyperkalemia is about one percent in ambulatory settings [1]. Its prevalence is higher among elderly patients in emergency departments (EDs) [2]. Given that hyperkalemia in hospitalized patients is associated with a higher mortality rate [3], It is crucial to establish an optimal treatment strategy for elderly patients with acute hyperkalemia in the acute care setting.

Treatment options to improve acute hyperkalemia include calcium salt, insulin with glucose, beta-agonists, bicarbonate, loop diuretics, potassium binders, and dialysis [4-9]. Few well-designed randomized trials have been conducted for most of these therapies [5]. Therefore, there is no evidence-based consensus about treatment strategies for acute hyperkalemia [5,6,9]. This results in variations of practice for acute hyperkalemia according to institutions [10]. Thus, it is crucial to gather evidence on therapies for acute hyperkalemia.

Potassium binders are pharmacological agents for acute hyperkalemia. Classically, sodium polystyrene sulfonate (SPS) and calcium polystyrene sulfonate (CPS) have been used as potassium binders for acute hyperkalemia to eliminate potassium from the body [5]. Sodium zirconium cyclosilicate (SZC) [11-13] and patiromer [14] have been recently approved as new potassium binders to improve hyperkalemia among ambulatory patients. Compared with traditional potassium binders such as SPS and CPS, SZC is a highly selective inorganic cation exchanger. It can decrease the serum potassium level by 0.5 mEq/L within four hours of administration [11]. Due to its immediate effect and few adverse effects, SZC has often been used for acute hyperkalemia in recent years.

Nonetheless, one pilot randomized placebo-controlled trial [15] to investigate the ability of SZC to improve hyperkalemia among ED patients reported that SZC did not significantly reduce the serum potassium level compared with placebo until more than four hours among ED patients with hyperkalemia. Moreover, several retrospective observational studies reported that SZC was similar to other potassium binders, such as SPS and CPS, in improving acute hyperkalemia [16-19]. Further studies are needed to determine the role of SZC in acute hyperkalemia. In addition, no such studies have been conducted in Japan, and few studies have focused on the elderly population. Given the aging-related changes in gut clearance [20] and the multi-comorbidities of elderly individuals [21], investigations into the efficacy and safety of SZC and CPS for elderly individuals are important. Therefore, the primary aim of the present study was to evaluate the comparative efficacy of SZC and CPS in reducing serum potassium levels in elderly patients with acute hyperkalemia and to assess the safety profile, particularly the risk of hypokalemia.

## Materials and methods

Study setting and design

This was a retrospective observational study to investigate the efficacy of SZC compared with CPS for reducing serum potassium in hospitalized elderly patients within 24 hours after administration. The data came from the electronic medical records (EMRs) of the National Hospital Organization (NHO) Tokyo Medical Center. The NHO Tokyo Medical Center is a 550-bed community acute care hospital. Our hospital provides emergency care to approximately 7000 patients who arrive by ambulance annually. However, this study design is limited by several forms of bias due to the lack of randomization and prospective data collection.

The protocol of this study was approved by the Medical Ethics Committee of the NHO Tokyo Medical Center (R23-091). We followed the Ethical Guidelines for Epidemiological Research in Japan and the Declaration of Helsinki. The need for individual informed consent was formally waived by the Medical Ethics Committee of the NHO Tokyo Medical Center because we collected deidentified data without contacting the patients. However, per the Japanese Ethical Guidelines, we displayed an opt-out statement in the waiting room and on the hospital’s webpage to inform the patients of the study and provide the opportunity to refuse the use of data.

Inclusion and exclusion criteria

All consecutive patients aged 65 years or older who were hospitalized at our hospital and who were newly prescribed SZC or CPS between January 2020 and September 2023 were included. This study period was chosen because SZC became available in Japan in 2020. Only patients with hyperkalemia, which was defined as serum potassium greater than 5.0 mEq/L before starting either of these drugs, were included. We excluded patients who underwent chronic dialysis therapy or who started dialysis within 24 hours after starting these drugs. We also excluded patients whose blood sample was not taken within 24 hours after starting these drugs because the primary aim was to investigate the change in serum potassium within 24 hours after potassium binder administration. Therefore, patients with non-urgent hyperkalemia without the need to monitor serum potassium levels within 24 hours were excluded in the present study..

Data collection

Information on demographic features, past medical history, regular medications, laboratory tests, treatments after admission, and prognosis was extracted from the EMRs. Before collecting the data, the two investigators (SY and JK) discussed the methods used to abstract information from the EMRs and made an Excel (Microsoft Corporation, Redmond, USA) spreadsheet for the data collection. From February 2024 to April 2024, the two investigators independently extracted all the data into the same Excel spreadsheet. They resolved any discrepancies through discussion. Any discrepancies were resolved through discussion between them.

Outcome measures

The primary outcome of this study was the change in serum potassium from the baseline value to the follow-up value closest to 24 hours after potassium binder administration. The secondary outcome was the proportion of patients whose serum potassium level was normalized until 24 hours after potassium binder administration. Another secondary outcome was hypokalemia, which was defined as a serum potassium level of less than 3.5 mEq/L within seven days after potassium binder administration.

Statistical analysis

The data of the study population are reported as descriptive statistics. The primary and secondary outcomes were compared between the patients who were prescribed SZC and those who were prescribed CPS by the chi-squared test or Mann‒Whitney U test. In post hoc analysis, the comparison between the two groups for the primary outcome was performed by using only data from patients who were not treated with insulin combined with glucose. These analyses were performed using Stata version 15 (LightStone, Tokyo, Japan) and BellCurve for Excel version 4.04 (Social Survey Research Information Co., Ltd., Tokyo, Japan).

## Results

**Figure 1:**
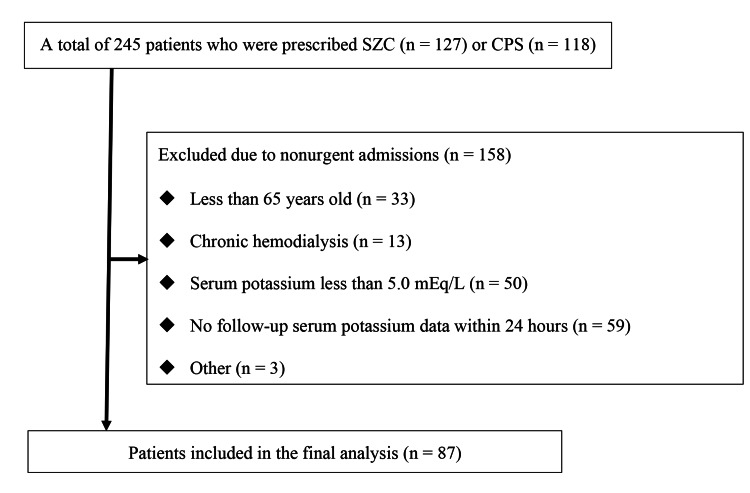
Flow chart of the 87 patients included in this study. CPS, calcium polystyrene sulfonate; SZC, sodium zirconium cyclosilicate.

During the study period, 245 hospitalized patients were newly prescribed SZC or CPS. Of those, 158 patients were excluded for the following reasons: no follow-up serum potassium data within 24 hours (n = 59), no hyperkalemia (n = 50), aged less than 65 years (n = 33), use of chronic hemodialysis (n = 13), and other conditions (n = 3). The other 87 patients were included in the final analysis (Figure 1).

The median age of the enrolled patients was 81 years (interquartile range (IQR) 75 to 86), 23 (26.4%) were women, 64 (73.6%) had chronic kidney disease, 32 (36.8%) had diabetes mellitus, and 22 (25.3%) had chronic heart failure (Table 1). The most common reasons for admission were acute kidney injury (n = 22, 25.3%), followed by pneumonia (n = 13, 14.9%) and heart failure (n = 7, 8.1%). Patient characteristics were similar between groups regarding age, comorbidities, vital signs, and baseline laboratory findings. The patients in the CPS group had hypertension more frequently than those in the SZC group, while pneumonia was a more common reason for admission in the SZC group. Both the median heart rate and potassium level at baseline were significantly higher in the SZC group than in the CPS group.

**Table 1:** Characteristics of the 87 patients with acute hyperkalemia. ^a^Comparisons between the SZC and CPS groups were conducted by the chi-squared test or Mann‒Whitney U test. CPS, calcium polystyrene sulfonate; IQR, interquartile range; SD, standard deviation; SZC, sodium zirconium cyclosilicate.

	Total (n = 87)	SZC group (n = 52)	CPS group (n = 35)	P value^a^
Demographic features				
Median age, years (IQR)	81 (75–86)	80 (74–85)	84 (78–88)	0.09
Men, n (%)	64 (73.6)	38 (73.1)	26 (74.3)	0.90
Median body mass index (IQR)	21 (19–24)	21 (19–24)	21 (19–24)	0.92
Past medical history, n (%)				
Chronic kidney disease	64 (73.6)	37 (71.2)	27 (77.1)	0.53
Diabetes mellitus	32 (36.8)	19 (36.5)	13 (37.1)	0.95
Hypertension	60 (69.0)	31 (59.6)	29 (82.9)	0.02
Heart failure	22 (25.3)	15 (28.9)	7 (20.0)	0.35
Regular medications, n (%)				
Loop diuretics	31 (35.6)	18 (34.6)	13 (37.1)	0.81
Mineralocorticoid receptor antagonists	13 (14.9)	8 (15.4)	5 (14.3)	0.89
Renin angiotensin system inhibitors	45 (44.6)	22 (42.3)	18 (51.4)	0.40
Neprilysin inhibitors	2 (2.3)	2 (3.9)	0 (0.0)	0.24
Beta-blockers	24 (27.6)	17 (32.7)	7 (20.0)	0.19
Insulin	14 (16.1)	7 (13.5)	7 (20.0)	0.42
Department, n (%)				
Emergency Medicine	15 (17.2)	12 (23.1)	3 (8.6)	0.08
Reasons for admission, n (%)				
Acute kidney injury	22 (25.3)	12 (23.1)	10 (28.6)	0.56
Pneumonia	13 (14.9)	11 (21.2)	2 (5.7)	0.047
Heart failure	7 (8.1)	4 (7.7)	3 (8.6)	0.88
Laboratory tests at baseline, median (IQR)				
Blood urea nitrogen, mg/dL	49.5 (34.6–70.3)	52.4 (34.8–85.3)	45.9 (34.6–62.7)	0.23
Creatinine, mg/dL	2.3 (1.4–3.6)	2.8 (1.5–3.8)	1.8 (1.4–3.3)	0.23
Sodium, mEq/L	136 (132–140)	135 (131–139)	137 (133–141)	0.34
Chloride, mEq/L	104 (98–108)	102 (97–107)	105 (98–109)	0.26
Bicarbonate, mEq/L	21.3 (18.3–25.2)	21.3 (18.4–25.1)	21.4 (16.6–25.5)	0.99
pH	7.36 (7.28–7.41)	7.36 (7.28–7.41)	7.35 (7.28–7.39)	0.83
ECG findings at baseline, n (%)				
Bradycardia	3 (3.5)	1 (1.9)	2 (5.7)	0.34
Tented T wave	10 (11.5)	6 (11.5)	4 (11.4)	0.99
Wide QRS	0 (0.0)	0 (0.0)	0 (0.0)	1.00

About 80% of patients in the SZC group received 30 grams per day of this drug, while no patients in the CPS group received more than 15 grams per day of the drug (Table 2). For the concomitant therapies, insulin with glucose, calcium gluconate, and loop diuretics were used in 40 (46.0%), 24 (27.6%), and 19 (21.8%) of all patients, respectively. Insulin combined with glucose was more frequently used in the SZC group than in the CPS group (p < 0.001).

**Table 2:** Treatment of acute hyperkalemia in the two groups. ^a^Includes only treatments used within 24 hours after administration of SZC or CPS. ^b^Comparisons between the SZC and CPS groups were conducted by the chi-squared test or Mann-Whitney U test. CPS, Calcium polystyrene sulfonate; N.A., not available; SZC, sodium zirconium cyclosilicate.

Treatment^a^, n (%)	Total (n = 87)	SZC group (n = 52)	CPS group (n = 35)	P-value^b^
Dose of SZC per day				
30 grams	41 (47.1)	41 (78.9)	0 (0.0)	N.A.
Less than 30 grams	11 (12.6)	11 (21.1)	0 (0.0)	N.A.
Dose of CPS per day				
15 grams	30 (34.5)	0 (0.0)	30 (85.7)	N.A.
Less than 15 grams	5 (5.7)	0 (0.0)	5 (14.3)	N.A.
Insulin with glucose	40 (46.0)	32 (61.5)	8 (22.9)	< 0.001
Calcium gluconate	24 (27.6)	20 (38.5)	4 (11.4)	0.01
Beta 2 receptor agonist	1 (1.2)	1 (1.9)	0 (0.0)	0.41
Bicarbonate therapy	4 (4.6)	2 (3.9)	2 (5.7)	0.68
Loop diuretics	19 (21.8)	12 (23.1)	7 (20.0)	0.73

For the primary outcome, the median changes in serum potassium from baseline to 24 hours after administration of the cation exchanger were -1.1 mEq/L and -0.5 mEq/L, respectively (Table 3). The reduction of serum potassium was significantly larger in the SZC group than in the CPS group (p = 0.003). Because insulin combined with glucose was more frequently administered to patients in the SZC group than to those in the CPS group, an additional analysis, which excluded patients who received insulin with glucose for acute hyperkalemia, was also conducted. In this post hoc analysis, there was no significant difference in the primary outcome between the SZC and CPS groups (-0.7 mEq/L versus -0.4 mEq/L, p = 0.39), although the significance of this result was limited due to the very small sample size in this study. Hypokalemia occurred in 12 patients (13.8%). All hypokalemic events were asymptomatic and occurred after 24 hours of administration of the study drugs. Hypokalemia was more common in the SZC group (n = 11, 21.2%) than in the CPS group (n = 1, 2.9%) (p = 0.02). In-hospital mortality was similar between the two groups.

**Table 3:** Comparison between the primary and other outcomes between the two groups. ^a^Comparisons between the SZC and CPS groups were conducted by using the chi-squared test or Mann‒Whitney U test. ^b^Hypokalemia was defined as a serum potassium level less than 3.5 mEq/L. CPS, calcium polystyrene sulfonate; IQR, interquartile range; SZC, sodium zirconium cyclosilicate.

Outcomes	Total (n = 87)	SZC group (n = 52)	CPS group (n = 35)	P value^a^
Median serum potassium level, mEq/L (IQR)				
At baseline	6.0 (5.7–6.4)	6.2 (5.8–6.4)	5.9 (5.6–6.2)	0.03
After 24 hours	5.1 (4.9–5.5)	5.0 (4.7–5.4)	5.3 (5.1–5.6)	0.04
Normalization of hyperkalemia within 24 hours, n (%)	38 (43.7)	29 (55.8)	9 (25.7)	0.01
Median change of serum potassium level within 24 hours, mEq/L (IQR)	0.8 (0.4–1.2)	1.1 (0.6–1.3)	0.5 (0.3–0.9)	0.003
Hypokalemia^b^ within one week, n (%)	12 (13.8)	11 (21.2)	1 (2.9)	0.02
In-hospital death, n (%)	17 (19.5)	10 (19.2)	7 (20.0)	0.93

## Discussion

Compared with CPS, SZC lowered the serum potassium level among elderly patients with acute hyperkalemia. However, hypokalemia was significantly more common in the SZC group than in the CPS group. Ours is the second study to compare the effects of SZC and CPS on lowering the serum potassium level among patients with acute hyperkalemia in an acute care setting.

The above results are not consistent with recent similar studies [16-19] showing that the effect of SZC on acute hyperkalemia in the acute care setting was similar to that of CPS or SPS. Several things could explain this discrepancy. First, the daily dose of SZC used in recent studies [16-19] is lower than the dose we used: 10 to 20 grams per day [16-19] vs. 30 grams in most cases in our study. Given that the ability of SZC to reduce the serum potassium concentration is dose-dependent [11,12], the use of lower doses of SZC in those studies might have resulted in a lower effect of SZC in previous studies [16-19] than in our study. Second, the use of insulin with glucose as concurrent therapy for acute hyperkalemia was more common in the SZC group than in the CPS group. Therefore, the efficacy of SZC at lowering the serum potassium level may have been overestimated in the present study. In fact, our post hoc analysis, which included only patients without insulin with glucose as a concurrent therapy, showed no difference in the efficacy of SZC and CPS at lowering serum potassium, although this analysis was post hoc and had a small sample size. Third, the dose of CPS used in the present study was lower than that used in previous studies [16]. Given that the effect of CPS on lowering serum potassium is dose-dependent [22], the present study might have underestimated the effect of CPS compared with a previous study [16]. Finally, our study focused only on elderly patients, in contrast to previous studies [16-19]. Thus, further studies are needed to evaluate whether SZC is superior to CPS for improving acute hyperkalemia in acute care settings. Although some experts recommend the use of SZC rather than SPS or CPS for acute hyperkalemia in the acute care setting [4], before the routine use of SZC for acute hyperkalemia, more evidence for the role of potassium binders in acute hyperkalemia to support this recommendation is needed.

In the present study, hypokalemia occurred in a substantial proportion of patients who received SZC, although there were no hypokalemic events within 24 hours after SZC administration. Hypokalemic events due to the use of SZC for ambulatory patients with hyperkalemia have rarely been reported in past randomized controlled trials [11-13,15]. Moreover, previous studies investigating the efficacy and safety of SZC for acute hyperkalemia [18,19,23] also reported that hypokalemia due to SZC was rare, although these studies evaluated only hypokalemic events within 24 hours after treatment initiation and involved lower SZC doses than those in the present study. Our results indicate that if SZC is used at the maximal dose, strict monitoring for hypokalemia due to SZC until one week after its initiation might be important in elderly patients with acute hyperkalemia.

Our findings imply that compared with the use of CPS, the use of higher SZC doses for elderly patients might improve acute hyperkalemia more effectively but may increase the risk of adverse events such as hypokalemia. Given that all hypokalemic events were asymptomatic in the present study, higher SZC doses with strict serum potassium monitoring might be an effective strategy for elderly patients with acute hyperkalemia. Further studies are needed to investigate the efficacy and safety of higher SZC doses in elderly patients hospitalized with acute hyperkalemia.

Several limitations should be mentioned. First, the present study was a retrospective observational study. Therefore, abstracting the data might not be accurate. Second, the single-center design limits the generalizability of our results. Third, the sample of our study was small. Finally, the present study included only elderly individuals. 

## Conclusions

Compared with CPS, SZC might significantly lower serum potassium in hospitalized elderly patients with hyperkalemia. Hypokalemia was more common in patients who received SZC than in those who received CPS. However, these findings were limited due to the small sample size and retrospective observational design of the present study. Therefore, further large randomized controlled studies are needed to determine the role of SZC in elderly hospitalized patients with acute hyperkalemia.
